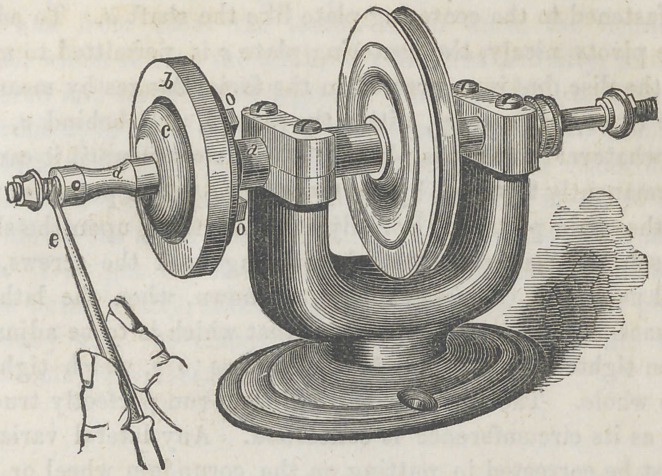# Saint John’s Adjustable Dental Lathe

**Published:** 1859-11

**Authors:** 


### SAINT JOHN'S ADJUSTABLE DENTAL LATHE.

The above cut represents an adjustable dental lathe, invented by St. John, of Bellefontaine, Ohio. The principle is good and very simple withal. The object is to adjust a bur or stone, or anything to be turned, to run perfectly true, though it may be eccentric upon the shaft. The mandrel *a* is adjusted upon a head and bearings of an ordinary structure; upon one end of the mandrel are collars and a screw, to attach a stone or brush, upon the other end is the adjustable center, which constitutes the principal peculiarity of this lathe. It consists of the circular plate, or disc *b*, attached firmly to the mandrel *a*, having on its face an annular depression, which receives the centering plate, *c*, which is about one-fourth of an inch less in diameter than the depression. To this centering plate is attached the shaft *d*, prepared for receiving upon its point a corundum wheel, or bur. There are four centering shafts accompanying each lathe, to which may be attached brushes, corundum wheels, burs and saws. There is a small chuck, denominated the watch-maker's pivoting chuck, which may be used by the dentist for holding fine drills, etc; it is fastened to the centering plate like the shaft *a*. To adjust the pivots nicely, the centering plate *c* is permitted to move in the disc, but is so pressed in the facial flanges by means of the two spiral springs, within the plate *b*, and behind *c*, that to whatever point it is placed, it will remain until it can be permanently fastened, by the two screws, in the posterior part of the main plate, *b*. For adjusting anything upon the shaft, loosen the centering plate by turning back the screws, *o o*, and using the centering tool, *e*, as shown, when the lathe is in motion, upon the periphery of that which is to be adjusted, then tighten by turning in the screws *o o*, which tightens the whole. The wheel or bur will then run perfectly true, so far as its circumference is concerned. Any lateral variation must be corrected in putting on the corundum wheel or bur. This lathe is a very valuable addition to the dentist's laboratory, and one that none should be without. It may be attached to any ordinary lathe. T.

## Figures and Tables

**Figure f1:**